# Greening of coffee waste through its transformation into clean and structurally stable activated carbon for energy storage applications

**DOI:** 10.1039/d5na00658a

**Published:** 2025-10-23

**Authors:** Zain Ul Abideen, Rasoul Khayyam Nekouei, Mohsen Hajian-Foroushani, Samane Maroufi, Veena Sahajwalla

**Affiliations:** a Centre for Sustainable Materials Research and Technology (SMaRT), School of Materials Science and Engineering, UNSW Sydney 2052 NSW Australia zain.abideen@unsw.edu.au

## Abstract

The sustainable transformation of biomass waste into high-purity activated carbon (AC) offers a promising solution to environmental challenges while advancing material innovations. However, conventional methods often yield materials with limited purity and performance. This study presents a scalable process to produce high-purity AC (99.7 wt%) from spent coffee grounds using optimized alkaline infusion, acid etching, and thermal treatment. The process achieves near-complete removal of inorganic residues and metal oxides, significantly improving structural, compositional, and electrochemical properties. Alkaline infusion with a 1 : 1.5 biochar-to-NaOH ratio reduced ash content from ∼7.8 to <0.3 wt% and increased carbon content to 83.6 wt%. Acid etching further removed residual oxides such as potassium and sodium, achieving 99.7 wt% purity. The surface area increased from 1.5 m^2^ g^−1^ (biochar) to 550 m^2^ g^−1^ (purified AC), with well-developed micropores (∼1.6 nm) and mesopores. As a proof of concept, the purified AC achieved a specific capacitance of 49 F g^−1^ at 0.5 A g^−1^, 59.6% higher than commercial AC (30.7 F g^−1^). Further thermal treatment at 1500 °C reduced oxygen content to 1.5 wt% while increasing carbon to 98.5 wt%. These results establish coffee waste-derived AC as a scalable, high-performance alternative for energy storage and circular economy applications.

## Abstract 2

Spent coffee waste was upcycled into high-purity activated carbon (99.7 wt%) with 59.6% higher capacitance, showcasing a scalable green route for sustainable energy storage and circular economy solutions.

## Graphical abstract

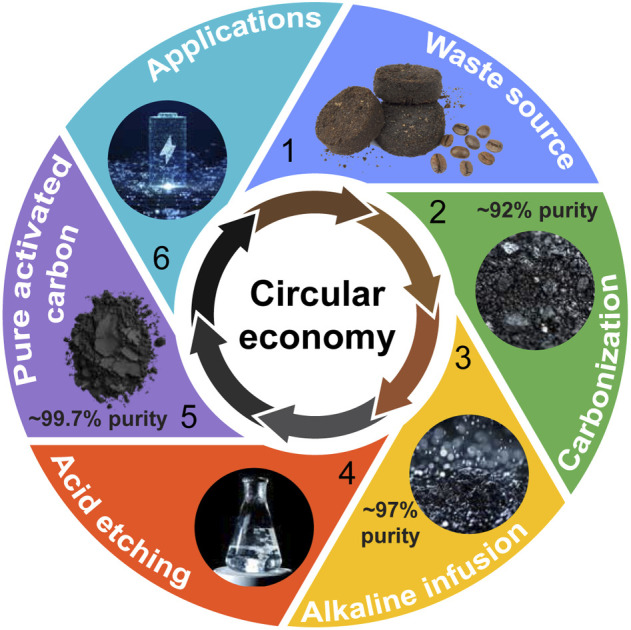

## Introduction

The global demand for activated carbon (AC) is projected to exceed 4 million tons annually by 2025, driven by its essential applications in water purification, air filtration, energy storage, and catalysis.^1–3^ The performance of AC in these applications is intrinsically linked to its physicochemical properties, such as surface area, pore size distribution, and purity.^4^ High-purity AC, characterized by an ash content below 0.5 wt%, is especially critical for advanced applications, including supercapacitors and batteries, where even minimal impurities can adversely affect electrochemical stability and efficiency.^5^ Despite its importance, achieving such high purity is a formidable challenge, particularly when using biomass waste as a precursor.^6^

Biomass-derived AC has emerged as a promising sustainable and economical alternative to conventional coal- or petroleum-based AC due to the abundance, renewability, and circular economy compatibility of biomass sources such as coconut shells, rice husks, and agricultural residues.^7–9^ However, the inherent composition of biomass often results in elevated ash content, typically ranging from 5 to 20 wt% in untreated AC or carbonized biomass, limiting its utility in high-purity and advanced applications.^10–12^ The effective removal of impurities, including inorganic residues and metal oxides, is critical to enhancing AC's performance in such high-value applications but presents a significant technical challenge.^12^

Chemical and thermal purification methods have been widely explored to address the impurity challenges in biomass-derived AC.^13^ Acid washing, in particular, is recognized as a highly effective technique for removing inorganic impurities, including metal oxides, silicates, and ash-forming compounds.^13,14^ Optimized acid treatments have successfully reduced ash content from 10 to below 1 wt% in AC derived from biomass sources such as rice husks and coconut shells.^15,16^ The choice of acid, its concentration, and treatment parameters significantly influence the outcome, as overly aggressive acid treatments can result in excessive carbon loss, reducing the AC yield to as low as 40–60%, depending on the precursor and processing conditions.^15,17^ Striking a balance between impurity removal and carbon retention remains a focal point in optimizing AC production.

Spent coffee ground, an underutilized but abundant agricultural residue, represents a promising precursor for AC production, contributing approximately 10 million tons of global waste annually.^18^ Its organic content makes it suitable for pyrolysis, yet its complex composition, including lipids, proteins, and inorganic residues, poses significant challenges for achieving high-purity AC.^19^ Unprocessed coffee-waste-derived AC typically exhibits ash content between 5 and 15 wt% and surface areas ranging from 300 to 800 m^2^ g^−1^.^19^ While such properties may suffice for basic applications such as filtration, they fall short for high-performance applications including energy storage and catalysis, emphasizing the need for effective purification strategies.

Alkaline treatments using NaOH or KOH have demonstrated substantial potential for improving the textural properties of AC while facilitating the removal of certain impurities.^20,21^ For instance, studies on AC derived from coconut shells and bamboo have shown that integrating alkaline treatment with thermal activation can achieve surface areas exceeding 1000 m^2^ g^−1^ and ash content below 0.5 wt%.^17,22^ When combined with acid washing, these methods further enhance purity by removing stubborn inorganic residues while preserving the structural integrity of the carbon matrix.^13,14,22^

However, similar integrated approaches have not been extensively applied to spent coffee ground, which presents greater compositional complexity than coconut shells or bamboo due to its high lipid and protein content and elevated alkali/alkaline earth oxides. Whereas coconut- and bamboo-derived AC can routinely achieve ash levels below 0.5 wt% and surface areas above 1000 m^2^ g^−1^ with moderate treatment,^17,22^ untreated coffee-waste-derived carbons typically contain 5–15 wt% ash and require more intensive purification to approach comparable purity.^19^ In addition, coffee grounds are globally abundant at ∼10 million tons annually but are underutilized, making their valorization not only technically challenging but also environmentally significant. Our process addresses these unique difficulties by combining NaOH infusion, acid etching, and thermal treatment, achieving 99.7 wt% carbon purity at 550 °C with reduced chemical input compared to conventional multi-step activations, thereby lowering energy consumption and processing cost while enabling high-performance AC production.

Recent advances have highlighted the importance of tailoring process parameters to achieve both high purity and desirable physicochemical properties in biomass-derived AC. For example, AC derived from corn husks has reached a purity level of 99.2 wt%, a surface area of 1100 m^2^ g^−1^, and a micropore volume of 0.35 cm^3^ g^−1^ through a combination of NaOH and HCl treatment.^22^ Such results highlight the effectiveness of integrating alkaline and acid treatments in addressing the dual challenge of impurity removal and pore optimization. However, spent coffee ground presents unique compositional and structural challenges that necessitate further exploration of scalable and efficient purification techniques.

**Fig. 1:**
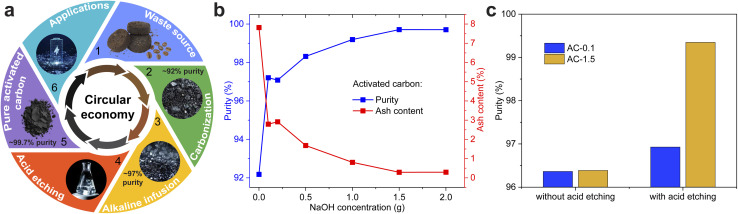
(a) Sequential process for producing high purity activated carbon from spent coffee ground, including pyrolysis, activation, and acid etching, achieving a final purity of ∼99.7 wt%. (b) Purity and ash content analysis of biochar and AC samples after alkaline infusion. (c) Purity analysis of AC-0.1 and AC-1.5 with and without acid etching.

In this study, we address the critical challenge of producing high-purity AC from spent coffee ground through a combination of alkaline infusion, acid etching, and thermal treatment. The methodology, illustrated in Fig. 1(a), achieved a purity level of 99.7 wt%, reduced ash content to below 0.3 wt%, and developed a high surface area of 550 m^2^ g^−1^ with well-distributed micropores and mesopores. As a proof of concept, the electrochemical performance of purified AC was evaluated in supercapacitor applications, achieving a specific capacitance of 49 F g^−1^ at a current density of 0.5 A g^−1^, outperforming commercial AC by 59.6%. By transforming spent coffee ground into high-purity AC with superior properties, this work contributes to advancing sustainable material production and resource recovery, addressing both environmental challenges and technological demands.

## Results and discussion

Table 1 outlines the sample notations and preparation conditions used in this study. Biochar, a carbon-rich solid produced through pyrolysis of biomass under limited oxygen, was derived from spent coffee grounds in this study, without chemical activation. Purified AC samples (AC-0.1 to AC-2) were prepared by infusing biochar with varying amounts of NaOH, with the number following “AC”- representing the grams of NaOH used during alkaline infusion experiments as described in the Experimental details section. These variations were designed to evaluate the impact of NaOH concentration on key parameters such as purity, porosity, and performance of the resulting materials. A commercial activated carbon (AC-commercial) was included for benchmarking against industrial standards.

**Table 1:** Sample notations and corresponding preparation conditions for activated carbon materials. AC-*x* denotes activation with a NaOH-to-biochar(dry) mass ratio of *x* : 1; *e.g.*, AC-1.5 corresponds to 1.5 g NaOH per 1 g biochar

Sample notation	Preparation conditions
Biochar	Carbonized residue from spent coffee ground
AC-0.1	Activated with 0.1 g NaOH
AC-0.2	Activated with 0.2 g NaOH
AC-0.5	Activated with 0.5 g NaOH
AC-1.0	Activated with 1.0 g NaOH
AC-1.5	Activated with 1.5 g NaOH
AC-2.0	Activated with 2.0 g NaOH
AC-commercial	Commercial sample for comparative analysis

### Effect of NaOH infusion on purity and ash content

The effect of NaOH infusion on the purity and ash content of the samples is presented in Fig. 1(b). In this study, “purity” refers specifically to the carbon mass fraction determined by proximate/elemental analysis after removal of inorganic residues; it does not imply the complete absence of mineral phases. Conversely, ash content reflects the level of residual inorganic impurities.^23^ Biochar, without alkaline infusion, exhibited the lowest purity (∼92 wt%) due to the residual organic and inorganic compounds.^19^

As shown in Fig. 1(b), alkaline infusion with NaOH significantly improved the purity of the materials, even at minimal concentrations. An infusion of 0.1 g of NaOH (AC-0.1) increased purity from ∼92 wt% to ∼97 wt% (∼5.4% increase), demonstrating the effectiveness of alkaline infusion in impurity removal. Increasing NaOH concentrations up to 1.5 g further enhanced the purity, peaking at 99.7 wt%; beyond this point the purity did not improve, indicating a practical saturation of impurity removal under these conditions.^8,22^

The trends in ash content inversely mirrored those of purity. Biochar exhibited the highest ash content (∼7.8 wt%). Increasing the NaOH concentration significantly reduced the ash content after alkaline infusion, achieving below 0.3 wt% for AC-1.5. Beyond this point, the ash content stabilized, indicating the effective removal of most ash-forming impurities with 1.5 g of NaOH infusion.^22,24^ These results show that NaOH infusion to 1.5 g is sufficient to suppress ash-forming impurities to <0.3 wt%, with no measurable benefit at higher doses.^22,24,25^

**Fig. 2:**
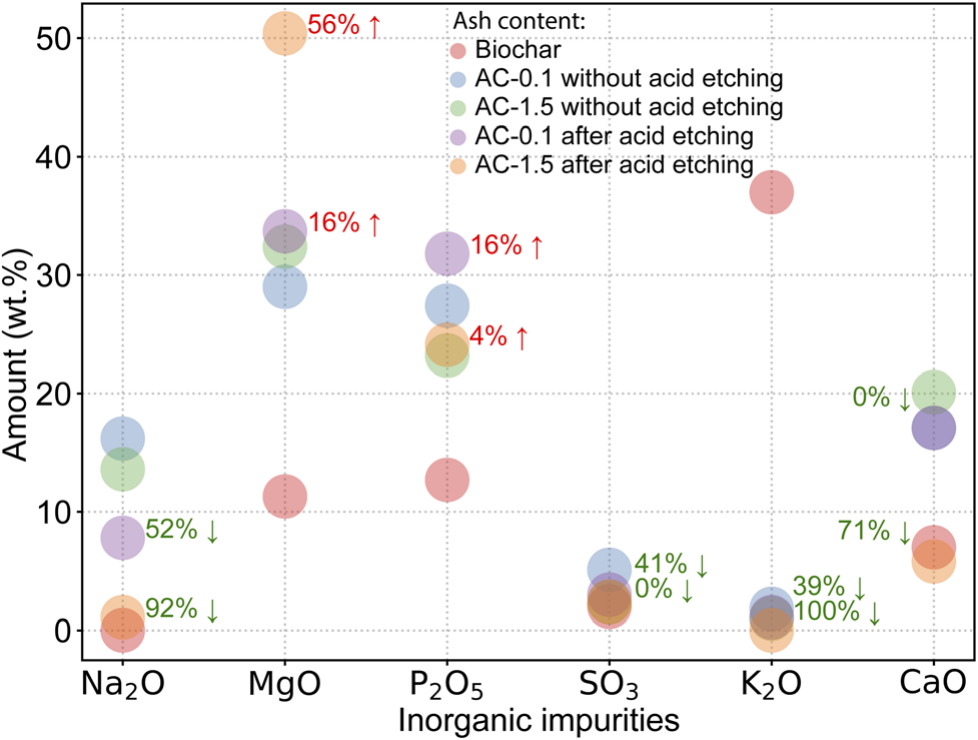
XRF analysis of key metal oxides in biochar and purified AC samples before and after acid etching, showing significant reductions in alkali oxides (K_2_O and Na_2_O). The percentages show percent change and the arrows direction show increase or decrease. Note: components below 0.5 wt% were excluded for clarity.

The alkaline infusion experiments were conducted at 550 °C in an argon atmosphere to prevent oxidation of the carbon matrix while facilitating impurity removal (see Experimental section). During the infusion process, NaOH reacts with oxide impurities in the spent coffee grounds, forming soluble compounds that are removed during subsequent water washing. The following are representative alkaline infusion reactions based on the detected impurities (SO_3_, MgO, P_2_O_5_, CaO, and K_2_O) in X-ray fluorescence (XRF) analysis of biochar prior to alkaline infusion (Fig. 2).^26^

1 CaO + 2NaOH + H_2_O → Ca(OH)_2_ + 2Na^+^

2 SO_3_ + 2NaOH + H_2_O → Na_2_SO_4_ + 2H_2_O

3 MgO + 2NaOH + H_2_O → Mg(OH)_2_ + 2Na^+^

4 P_2_O_5_ + 6NaOH + 3H_2_O → 2Na_3_PO_4_ + 3H_2_O

5 K_2_O + 2NaOH + H_2_O → 2KOH + 2Na^+^

These reactions demonstrate the role of NaOH in solubilizing oxide impurities, reducing ash content, and increasing the purity of AC.

### Effect of acid etching on purity

**Fig. 3:**
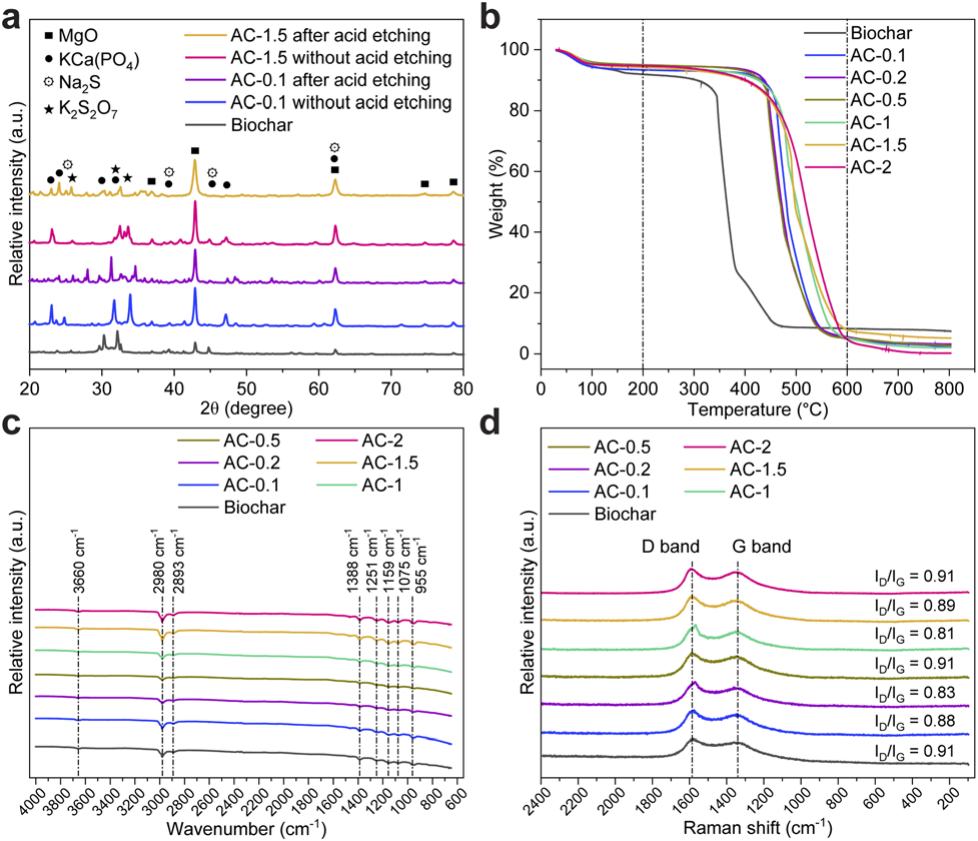
(a) XRD patterns of biochar and AC-0.1 and AC-1.5 with and without acid etching. (b) TGA curves, (c) FTIR and (d) Raman spectra of biochar and purified AC samples (AC-0.1 to AC-2.0).

The impact of acid etching as a post-alkaline infusion step is presented in Fig. 1(c). Acid etching complements alkaline infusion by targeting residual inorganic impurities, resulting in the production of high-purity carbon materials.^27^ For AC-1.5, the carbon mass fraction increased from ∼97 wt% before acid etching to 99.7 wt% afterwards, demonstrating effective removal of most ash-forming elements, however, residual MgO remains detectable in XRF/XRD (Fig. 2 and 3(a)), reflecting its partial resistance to acid leaching.

The XRF results in Fig. 2 illustrate the elemental composition changes during alkaline infusion and acid etching, demonstrating the purification and compositional refinement of the AC samples. As can be seen in Fig. 2, the content of potassium oxide (K_2_O), initially dominant in biochar at 37 wt%, reduced to 1.8 and 1 wt% in AC-0.1 and AC-1.5, respectively, after alkaline infusion. Acid etching further removed K_2_O, reducing it to 1.1 wt% in AC-0.1 and rendering it undetectable in AC-1.5, confirming the near-complete elimination of potassium residues. These findings align with previous studies on acid etching for alkali metal removal in biomass-derived AC. However, unlike conventional methods that often cause excessive carbon loss, this work optimizes the synergistic combination of alkaline infusion and acid etching to achieve 99.7 wt% purity while preserving structural integrity.^28^

Sodium oxide (Na_2_O), which was introduced during alkaline infusion, was also significantly reduced post-acid etching. In AC-0.1, Na_2_O decreased from 16.2 wt% to 7.8 wt%, while in AC-1.5, it dropped from 13.6 wt% to 1.1 wt%, representing a 91.9% reduction. Acid etching facilitated these reductions by dissolving inorganic impurities, such as potassium, sodium, calcium, and sulphur residues, through chemical reactions as shown below:^26^

6 K_2_O + 2HCl + H_2_O → 2KCl + 2H_2_O

7 Na_2_O + 2HCl + H_2_O → 2NaCl + 2H_2_O

8 MgO + 2HCl → MgCl_2_ + H_2_O

9 P_2_O_5_ + 4HCl + H_2_O → 2H_3_PO_4_ + 4HCl

10 CaO + 2HCl → CaCl_2_ + H_2_O

11 SO_3_ + 2HCl + H_2_O → H_2_SO_4_ + 2HCl

Magnesium oxide (MgO), although increasing in content during alkaline infusion, was relatively resistant to acid etching, enriching from 32.4 wt% to 50.4 wt% in AC-1.5. In contrast, calcium oxide (CaO) exhibited significant reductions post-acid etching, decreasing from 20.1 wt% to 5.8 wt% in AC-1.5, corresponding to a 71.1% reduction. Phosphorus pentoxide (P_2_O_5_) exhibited variable behaviour, stabilizing at 24.1 wt% in AC-1.5 after acid etching, reflecting its partial retention due to chemical interactions during infusion and acid etching processes.

The XRD analysis in Fig. 3(a) provide qualitative confirmation of the compositional changes observed in the XRF analysis, identifying key crystalline phases present in the residual ash of biochar and purified AC samples. The major detected phases, MgO, KCa(PO_4_), Na_2_S, and K_2_S_2_O_7_ present in unwashed samples, highlight the effectiveness of the purification process in eliminating most inorganic impurities after acid etching.

Biochar exhibited multiple crystalline peaks, reflecting its high ash content (∼7.8 wt%). Following NaOH infusion, peaks corresponding to Na_2_S and K_2_S_2_O_7_ were reduced in AC-0.1, suggesting their dissolution during alkaline treatment. However, KCa(PO_4_) and MgO remained detectable with MgO intensities increasing. This trend indicates their lower reactivity under alkaline conditions and a relative enrichment effect, as other impurities were removed. Subsequent acid etching further suppressed the KCa(PO_4_) and Na_2_S peaks in AC-0.1, confirming their removal through acid dissolution.

A similar trend was observed for AC-1.5, where alkaline infusion facilitated substantial impurity removal, yet MgO peaks intensified relative to untreated biochar. The persistence of MgO after acid etching is likely due to its limited solubility in HCl. While MgO reacts with HCl to form MgCl_2_, the formation of a passivating Mg(OH)_2_ layer inhibits complete dissolution. Studies have shown that MgO exhibits only partial solubility in acidic environments, with dissolution rates as low as 43% in simulated gastric acid.^29,30^ Furthermore, MgO may incorporate into stable mineral matrices, such as magnesium phosphates, reducing its accessibility to acid etching.^31^ This suggests that alternative purification strategies, such as stronger acid treatments, extended etching duration, or the use of chelating agents, may be required for complete MgO removal.^30,31^

### Thermal stability and composition analysis

The thermal stability and compositional characteristics of biochar and purified AC samples were analysed using thermogravimetric analysis (TGA) and derivative thermogravimetric (DTG) analysis, as shown in Fig. 3(b) and S1(a). Biochar exhibited a two-stage weight loss: an initial loss of 7 wt% below 150 °C, attributed to moisture evaporation, followed by a significant loss between 300–500 °C, corresponding to the decomposition of residual organic matter.^18,23^ This organic matter primarily consists of biopolymers such as cellulose, hemicellulose, and lignin, which are incompletely removed during initial pyrolysis. Under thermal treatment, these biopolymers decompose as heat cleaves their chemical bonds, resulting in the observed weight loss.^18,23^ The remaining weight (∼8 wt%) at 800 °C indicates thermally stable inorganic residues.

Purified AC, such as AC-0.1 and AC-1.5, demonstrated improved thermal stability, as indicated by thermogravimetric analysis. The initial weight loss of approximately 5 wt% below 150 °C corresponds to the removal of residual moisture. The major weight loss event, accounting for approximately 80% of total mass, occurs between 400–600 °C and is attributed to the decomposition of complex organic compounds present in the precursor material. Spent coffee ground contains a diverse range of biopolymers, including cellulose, hemicellulose, lignin, lipids, and proteins, which exhibit different thermal decomposition behaviours.

Hemicellulose, the least thermally stable component, decomposes between 250–350 °C, undergoing depolymerization and fragmentation, releasing volatile compounds such as CO_2_, CO, and small hydrocarbon species. Cellulose follows, decomposing in the 300–400 °C range through random scission and dehydration, leading to the breakdown of glycosidic linkages and the formation of tar-like intermediates. Lignin, the most thermally stable biopolymer, exhibits broad decomposition from 200–700 °C, with a gradual release of phenolic compounds, aromatics, and char residues, contributing to carbon yield.^18^

In this study, the alkaline infusion and acid etching processes play a crucial role in modifying the decomposition pathways of these organic compounds. NaOH infusion facilitates the selective breakdown of thermally labile components by catalysing the hydrolysis of ester and ether bonds, promoting the degradation of cellulose and hemicellulose while stabilizing the carbon framework. Acid etching removes residual inorganic contaminants, such as metal oxides and alkali salts, which can catalyse unwanted side reactions during thermal decomposition. This controlled processing results in a more stable carbon structure, reducing the formation of residual tars and non-carbonized fractions. For AC-1.5, the residual weight at 800 °C dropped below 5%, confirming the near-complete elimination of inorganic impurities and thermally unstable organic residues.^18,23^

The DTG profiles in Fig. S1(a) further illustrate the decomposition behaviour. Biochar displayed a broad and intense peak between 300–400 °C, linked to the breakdown of unstable organic groups and biomass residues.^32^ In contrast, purified AC samples showed shifted peaks to 400–600 °C, reflecting the removal of organic impurities and restructuring of the carbon matrix. AC-1.5 exhibited a pronounced peak around 500 °C, demonstrating its high purity and enhanced thermal stability. Minimal weight loss above 600 °C confirmed the effective removal of significant inorganic residues.

FTIR analysis (Fig. 3(c)) of biochar and purified AC samples revealed significant changes in surface chemistry due to NaOH infusion and acid etching. The prominent peak at 3660 cm^−1^, associated with O–H stretching vibrations from hydroxyl groups and adsorbed water, was substantially reduced in infused samples like AC-1.5, indicating the effective removal of hydrophilic functional groups, potentially contributing to increased hydrophobicity.^33,34^ The C–H stretching vibrations at 2980 cm^−1^ and 2893 cm^−1^, attributed to aliphatic hydrocarbons, were diminished or absent in infused samples, reflecting the decomposition of residual organic matter during alkaline infusion.^33,34^ Similarly, the symmetric COO^−^ stretching vibration at 1388 cm^−1^, linked to carboxylate groups, was significantly reduced in AC-1.5, demonstrating the efficient removal of carboxylic acid derivatives. Peaks corresponding to C–O stretching vibrations (1159 cm^−1^ and 1075 cm^−1^) also showed reduced intensity, highlighting a cleaner and oxygen-depleted carbon surface.^34,35^ The persistence of the aromatic C–H bending vibration at 955 cm^−1^ across all samples suggests the structural integrity and stability of aromatic carbon frameworks during chemical treatment.^35^

Raman spectroscopy (Fig. 3(d)) provided insights into the structural evolution of the samples. The G band (1585 cm^−1^) corresponds to sp^2^-hybridized graphitic carbon, while the D band (1350 cm^−1^) represents disordered carbon structures and defects. The intensity ratio of the D to G bands (*I*_D_/*I*_G_) quantifies graphitic order and defect density.^36,37^ Biochar exhibited an *I*_D_/*I*_G_ ratio of 0.91, indicative of substantial structural disorder and a high proportion of amorphous carbon due to incomplete pyrolysis. NaOH infusion improved structural order, as shown by a decrease in the *I*_D_/*I*_G_ ratio to 0.88 for AC-0.1, reflecting the removal of amorphous phases and enhanced graphitization. AC-1.5 displayed an *I*_D_/*I*_G_ ratio of 0.89, signifying reduced defect density and a balanced improvement in graphitization.^37,38^ However, AC-2 exhibited an *I*_D_/*I*_G_ ratio of 0.91, similar to biochar, suggesting that excessive NaOH infusion can degrade the graphitic structure or reintroduce defects.^38^

The combined TGA, FTIR, and Raman analyses highlight that NaOH infusion at 1.5 g (AC-1.5) achieves a balance of purity, thermal stability, and graphitic order. The significant reduction in oxygen-containing functional groups, coupled with a lower *I*_D_/*I*_G_ ratio and minimal ash content, demonstrates the efficacy of optimized chemical infusion and acid etching in producing high-quality AC.

### Elemental composition analysis

**Fig. 4:**
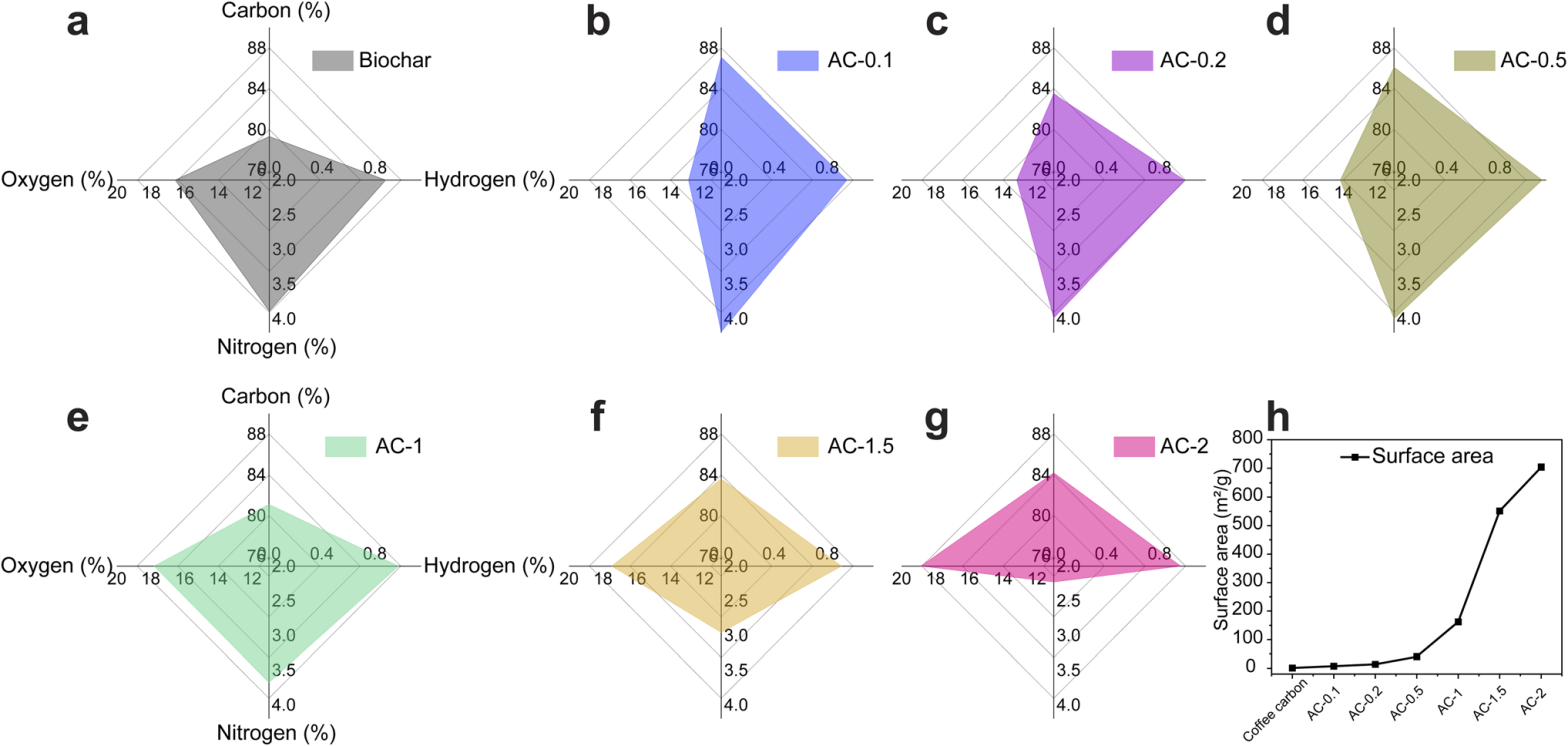
(a–g) Radar plots summarizing CHNS and oxygen analyses and (h) BET surface area results for biochar and AC samples. NaOH infusion improves carbon content and reduces oxygen, with AC-0.1 achieving the highest carbon content (87.1 wt%) and AC-1.5 balancing purity (83.7 wt%) and oxygen retention (17.6 wt%). BET surface area increases from 1.5 m^2^ g^−1^ for biochar to 550 m^2^ g^−1^ for AC-1.5.

The radar plots in Fig. 4(a)–(g) and the data in Tables S1 and S2, summarize the results of carbon, hydrogen, nitrogen, sulphur (CHNS) and oxygen analysis, illustrating the chemical transformations induced by NaOH infusion and acid etching. These results highlight the interplay between carbon content, functional group removal, and oxygen retention, all of which contribute to the tailored surface chemistry of biochar and purified AC.

Biochar exhibits a carbon content of 79.3 wt%, with relatively high nitrogen (3.9 wt%) and oxygen (16.7 wt%) levels, indicative of the incomplete removal of residual functional groups (Fig. 4(a)). Nitrogen in biochar originates from proteinaceous compounds and amino acids present in spent coffee ground which are partially retained during pyrolysis.^39^ The low hydrogen content (0.9 wt%) corresponds to limited hydrophobicity, as hydrophobicity is influenced by both the degree of surface oxygenation and carbon content.^40,41^ These characteristics reflect the unrefined structure of biochar, with significant potential for improvement through chemical treatments.

The chemical treatments significantly enhanced the elemental composition of the purified AC. AC-0.1 achieved the highest carbon content (87.1 wt%) and the lowest oxygen content (12.9 wt%), reflecting effective removal of oxygen-containing functional groups and a cleaner carbon matrix (Fig. 4(b)). AC-1.5 achieved a well-balanced composition, with a carbon content of 83.6 wt%, reduced nitrogen (2.9 wt%), and low hydrogen content (0.9 wt%), demonstrating its tailored surface properties and improved purity. The oxygen content (17.5 wt%) in AC-1.5 indicates the presence of surface functionalities beneficial for advanced applications, such as energy storage (Fig. 4(f)). In contrast, AC-2 exhibited the highest oxygen content (18.9 wt%), indicating the retention of oxygen-rich functionalities that enhanced surface reactivity. The nitrogen content decreased progressively across the series, with AC-2 retaining the least nitrogen (2.2 wt%), corresponding to minimal retention of nitrogen-containing groups.

### Surface area and porosity analysis

The BET surface area results in Fig. 4(h) highlight the transformation of biochar into porous, high-surface-area purified AC after alkaline infusion and acid etching. Surface area is a key parameter for adsorption capacity and functional performance in applications such as catalysis, energy storage, and environmental remediation.^42^ Biochar, with a surface area of only 1.5 m^2^ g^−1^, exhibits negligible porosity due to its dense structure and incomplete pyrolysis.

NaOH infusion significantly enhanced the surface area by promoting the formation of micropores and mesopores. Initial infusion with 0.1 g of NaOH increased the surface area to 7.8 m^2^ g^−1^, reflecting the early stages of porosity development through partial removal of amorphous carbon and surface impurities.^43–45^ As the NaOH concentration increased, the surface area raised to 14.4 m^2^ g^−1^ for AC-0.2, indicating progressive micropore formation. AC-0.5 exhibited a surface area of 40.7 m^2^ g^−1^, demonstrating substantial pore formation and enhanced surface accessibility. This trend continued with AC-1 (162 m^2^ g^−1^) and AC-1.5 (550 m^2^ g^−1^), the latter exhibiting a well-developed network of micropores and mesopores due to the complete removal of amorphous carbon and enhanced pore accessibility.^44,45^ Interestingly, AC-2 achieved the highest BET surface area (705 m^2^ g^−1^); however, pore width distribution analysis (Fig. S1(b)) shows that this increase arises mainly from ultra-micropores (<1 nm) with limited mesopore contribution. Such pores contribute strongly to gas adsorption (thus elevating BET values) but remain only partially accessible to solvated electrolyte ions. The lack of interconnected mesoporous channels restricts ion transport, which explains the relatively lower capacitance of AC-2 despite its nominally high surface area.^43,44,46^

Pore volume analysis in Fig. S1(b) provides further insights into porosity development. Biochar demonstrated minimal pore volume with a flat profile across pore width ranges, consistent with a dense, predominantly macroporous structure resulting from incomplete pyrolysis.^47^ In contrast, AC samples exhibited a marked increase in pore volume, particularly in the micropore (<2 nm) and mesopore (2–4 nm) ranges, highlighting the impact of NaOH infusion. AC-1 showed a distinct mesopore peak (∼2–3 nm) (Fig. S1(b)), reflecting new porosity formation though its total pore volume remained relatively low. This indicates that the infusion at this concentration is sufficient to initiate pore development but not extensive enough to create a highly porous structure. AC-1.5 attained a total pore volume of 0.019 cm^3^ g^−1^, indicative of continuous formation of micropores and mesopores, characterized by a dominant mesopore peak at ∼1.6 nm and broad contributions from micropores. This optimal balance maximized surface accessibility and adsorption capacity, making AC-1.5 particularly suitable for applications requiring both high surface area and well-developed porosity. The broad distribution of pore sizes in AC-2 (pore volume ∼ 0.024 cm^3^ g^−1^) reflects extensive infusion but the observed diminishing returns highlights the risk of oversaturation effects that can compromise structural integrity.^47,48^

The nitrogen adsorption–desorption isotherms in Fig. S1(d) illustrate the evolution of porosity during NaOH infusion. Biochar, AC-0.1, AC-0.2, and AC-0.5 exhibited Type II isotherms, characteristic of non-porous or macroporous materials. These samples showed minimal nitrogen adsorption, no evidence of micropore filling, and an absence of a hysteresis loop, consistent with their dense and relatively unrefined structures.^49,50^ In contrast, AC-1, AC-1.5, and AC-2 displayed Type IV isotherms, indicative of mesoporous materials.^49,50^ Steep nitrogen uptake at low relative pressures (*P*/*P*^0^ < 0.1) signifies initial micropore filling, while the presence of hysteresis loops at higher pressures (*P*/*P*^0^ > 0.4) confirms well-developed mesoporosity (Fig. S1(e)). AC-0.5, AC-1, and AC-1.5 showed significantly higher nitrogen uptake and well-defined hysteresis loops, reflecting the successful development of both microporosity and mesoporosity.^49^

### Morphological analysis

**Fig. 5:**
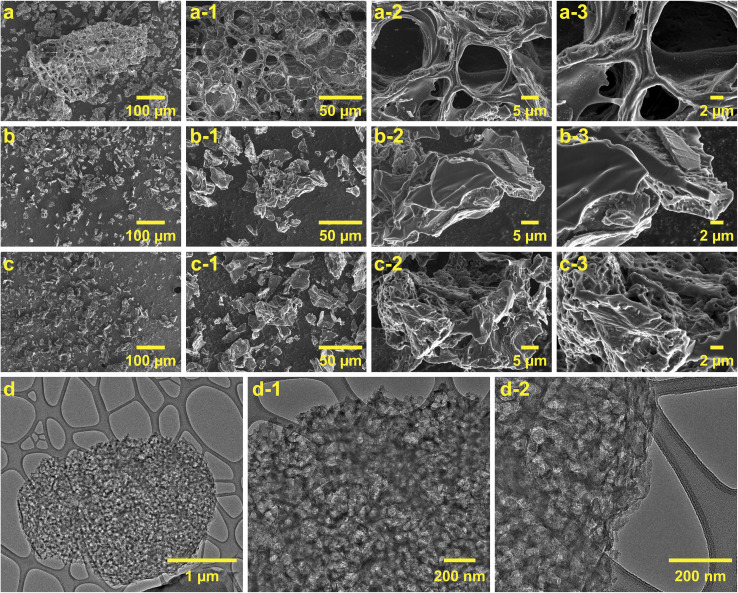
SEM and TEM images of biochar, AC-0.1, and AC-1.5. (a–a-3) Biochar shows macroporosity and a dense structure. (b–b-3) AC-0.1 displays initial micropore formation and surface irregularities. (c–c-3) AC-1.5 exhibits a well-developed, interconnected porous network. (d–d-2) TEM images of AC-1.5 highlight uniform micropore distribution and structural integrity.

Fig. 5 presents the SEM and TEM micrographs of biochar, AC-0.1, and AC-1.5, illustrating the morphological changes induced by alkaline infusion and acid etching.

The SEM images of biochar Fig. 5(a)–(a-3) reveal a relatively dense, compact structure dominated by significant macroporosity and limited microporosity, consistent with its low BET surface area and incomplete pyrolysis. The absence of micropores and mesopores suggests minimal removal of amorphous carbon and inadequate pore development during pyrolysis. This morphology aligns with the low adsorption capacity and negligible nitrogen uptake observed in the BET analysis, indicating a material with limited applicability for advanced functional uses.

In contrast, the SEM images of AC-0.1 Fig. 5(b)–(b-3) exhibit the emergence of initial micropores and mesopores, along with visible surface irregularities and openings. These morphological changes reflect the early stages of NaOH infusion, which facilitates partial removal of surface impurities and initiates shallow, non-interconnected micropores formation. However, the modest BET surface area and limited pore volume of AC-0.1 indicate that the structure remains underdeveloped at this stage. The SEM images of AC-1.5 Fig. 5(c)–(c-3) reveal a highly refined porous network characterized by significant surface roughness and interconnected micropores and mesopores. The formation of deeper, well-connected pores corresponds to the substantial increases in BET surface area and pore volume, demonstrating the effectiveness of the optimized alkaline infusion and acid etching processes.

The TEM images of purified AC-1.5 Fig. 5(d)–(d-2) provide further insights into its structural properties at higher resolutions. Fig. 5(d) shows the uniform distribution of micropores across the carbon matrix, while Fig. 5(d-1) and (d-2) highlight the interconnected voids and the well-preserved structural integrity of the pore network. These features corroborate the enhanced adsorption capacity and balanced distribution of micropores and mesopores observed in the pore volume analysis. Moreover, the combined effects of NaOH infusion and acid etching not only enhanced porosity but also facilitated the removal of impurities, resulting in the high purity of AC-1.5.

### Electrochemical characterization for energy storage applications

The high quality and high purity of AC are particularly valued for their significant potential in catalysis, environmental remediation, energy storage, and sensing technologies.^51–53^ To demonstrate the practical utility and feasibility of the regenerated high-purity AC derived from biomass waste, its electrochemical performance was evaluated in a supercapacitor application and compared against commercial AC. This comparative analysis highlights the potential of high-purity AC as a sustainable and effective alternative to commercially produced AC.

**Fig. 6:**
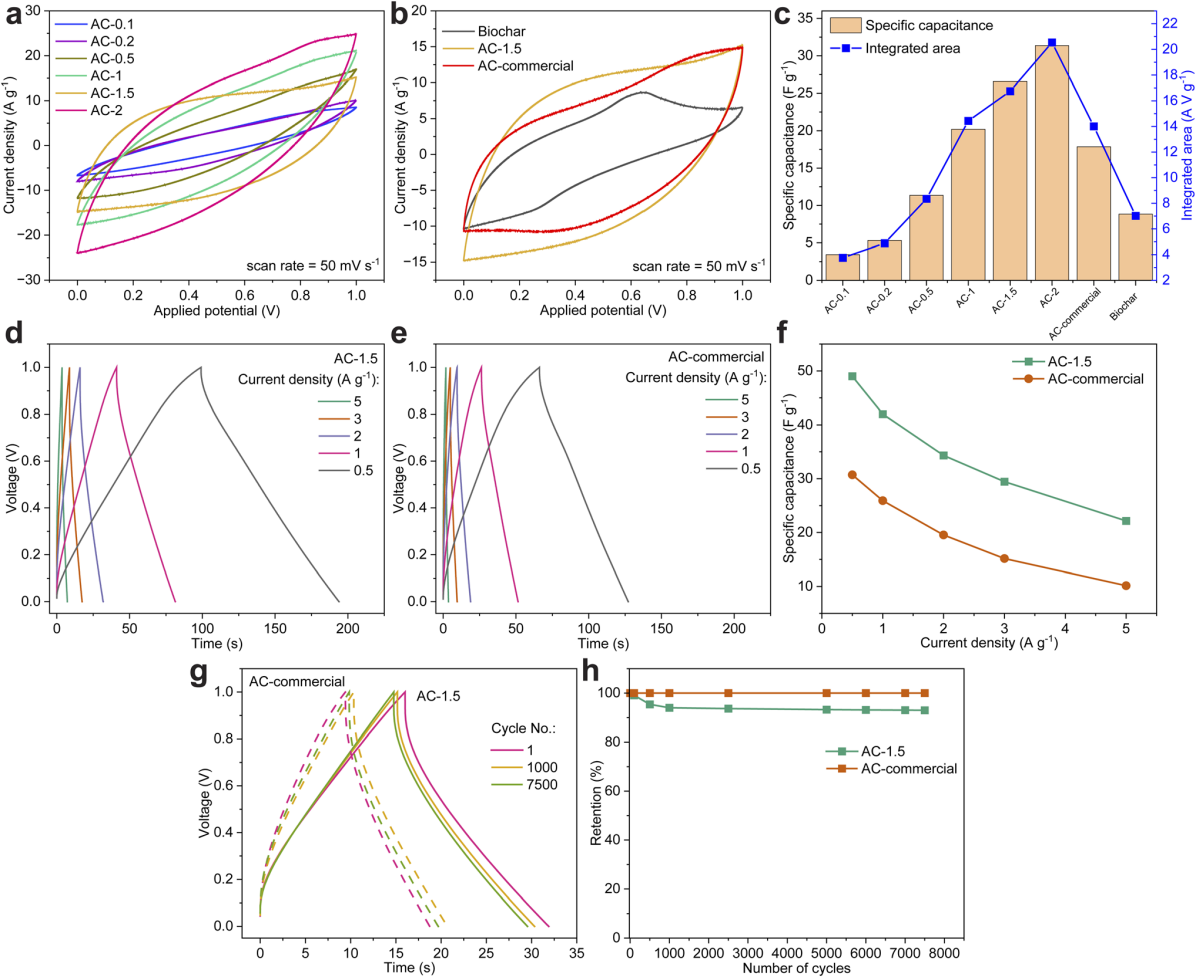
Electrochemical performance of biochar, AC-1.5, AC-commercial, and other purified AC samples. (a) CV curves at 50 mV s^−1^; the enclosed area under each CV curve was integrated to evaluate charge storage. (b) CV comparison of biochar, AC-1.5, and AC-commercial. (c) Specific capacitance (F g^−1^) and integrated CV curve area, showing AC-1.5 as representative. (d and e) Charge–discharge profiles of AC-1.5 and AC-commercial, achieving 49 F g^−1^ and 30.7 F g^−1^ at 0.5 A g^−1^, respectively. (f) Specific capacitance (F g^−1^) across current densities. (g) Cycling stability and (h) capacitance retention over 7500 cycles at 2 A g^−1^, with AC-1.5 retaining 93% and AC-commercial retaining 100% of their initial capacitance.

Fig. 6(a) presents the cyclic voltammetry (CV) curves of biochar and AC samples at a scan rate of 50 mV s^−1^, illustrating the enhanced electrochemical performance achieved through purification. Purified AC samples exhibited nearly symmetric CV curves, reflecting a combination of double-layer capacitance and pseudocapacitance, both of which are essential for supercapacitor applications.^23^ In contrast, biochar showed a narrow CV curve with minimal current response, which was attributed to its low surface area and limited active sites. The progressive expansion of the CV curves with increasing NaOH infusion highlights the improvements in charge storage capabilities, attributed to the enhanced pore structure and surface functionalities. Among the purified AC samples, AC-2.0 exhibited the most expansive CV curve, consistent with its higher BET surface area (705 m^2^ g^−1^); however, the surface-area-normalized capacitance *C*_A_ = *C*_sp_/SSA ≈ 4.4 μF cm^−2^ (31 F g^−1^/705 m^2^ g^−1^) indicates partial utilization of the created area at 50 mV s^−1^ (Fig. 6(a) and (c)).

Fig. 6(b) compares the CV curves of biochar, AC-1.5, and AC-commercial. The narrow CV curve of biochar reflected its poor charge storage capability stemming from its minimal surface area and the lack of porosity. In contrast, AC-1.5 exhibited a larger, nearly rectangular CV curve, indicating better charge storage capacity and high reversibility. The larger enclosed area of AC-1.5 compared to AC-commercial emphasizes its optimized pore structure and surface functionalities, which enhance ion transport and charge storage efficiency.

Fig. 6(c) compares the specific capacitance and integrated CV curve areas of biochar and purified AC samples, providing a quantitative assessment of their electrochemical performance. Biochar exhibited the lowest specific capacitance (8 F g^−1^) and integrated area (7 A V g^−1^). AC-0.1 and AC-0.2 demonstrated initial improvements in specific capacitance and integrated area, reflecting the early stages of pore development and surface activation. AC-0.5 achieved further enhancement, with specific capacitance and integrated area values of 11.3 F g^−1^ and 8 A V g^−1^, respectively. AC-1.0 showed a marked improvement with a specific capacitance of 20.2 F g^−1^ and an integrated area of 14.4 A V g^−1^.

AC-1.5 exhibited specific capacitance and CV-integrated charge of 26.6 F g^−1^ and 16.7 A V g^−1^, respectively, normalized by area this corresponds to *C*_A_ ≈ 4.8 μF cm^−2^ (26.6/550), indicating efficient utilization of accessible surface at 50 mV s^−1^ (Fig. 6(c)). AC-2.0 achieved the highest specific capacitance (31 F g^−1^) and integrated area (20.5 A V g^−1^), however, *C*_A_ ≈ 4.4 μF cm^−2^ is slightly lower than AC-1.5, consistent with a larger fraction of sub-2 nm pores that are rate-limited at this scan rate (Fig. 6(c)).

Fig. 6(d)–(f) compares the electrochemical performance of AC-1.5 and AC-commercial under varying current densities, offering insights into their rate capabilities and specific capacitance retention. The galvanostatic charge–discharge (GCD) profiles of AC-1.5 (Fig. 6(d)) in nearly triangular shapes with long discharge times at lower current densities is an indicative of good capacitive behaviour and minimal resistive losses. At 0.5 A g^−1^, AC-1.5 achieved a specific capacitance of 49 F g^−1^, outperforming AC-commercial, which recorded 30.7 F g^−1^ (Fig. 6(e) and (f)).

Although AC-2 reaches the highest specific capacitance (31 F g^−1^ at 50 mV s^−1^), its performance is hindered by limited ion accessibility due to ultra-micropore dominance (Fig. S1(b)) and higher *IR* drops at elevated current densities (Fig. S2(a)). In contrast, AC-1.5 combines a high capacitance (26.6 F g^−1^), lower polarization losses, and excellent cycling stability (93% retention after 7500 cycles, Fig. 6(h)), outperforming AC-2 in long-term reliability and rate capability. Moreover, AC-1.5 achieves this performance at a lower chemical input level (1.5 g NaOH per g biochar), reducing reagent consumption, minimizing secondary waste, and thereby offering a more sustainable processing route compared to AC-2. These combined features designate AC-1.5 as the most balanced, sustainable, and practically optimal material for supercapacitor applications. At a current density of 0.5 A g^−1^, AC-1.5 exhibited a 59.6% higher specific capacitance than AC-commercial, and even at 5 A g^−1^, it retained over double the specific capacitance. Across the tested current densities, AC-1.5 maintains higher gravimetric capacitance than AC-commercial (*e.g.*, 49 *vs.* 30.7 F g^−1^ at 0.5 A g^−1^) while exhibiting modest *IR*-drops (Fig. S2), indicating rapid electrolyte access to electrochemically accessible surface and stable double-layer storage under the employed conditions.

Fig. S2 compares the *IR* drop and resistance values of AC-1.5 and AC-commercial across different current densities, providing insights into their internal resistance and electrochemical performance. As can be seen in Fig. S2(a), AC-1.5 exhibited consistently higher *IR* drop values than AC-commercial, indicating greater internal resistance. At 0.5 A g^−1^, the *IR* drop (equivalent series resistance, ESR) for AC-1.5 is 15 mV compared to 11 mV for AC-commercial. This trend persisted at higher current densities, with AC-1.5 reaching 151 mV at 5 A g^−1^, while AC-commercial records 111 mV. The higher *IR* drop of AC-1.5 (15 mV at 0.5 A g^−1^; 151 mV at 5 A g^−1^) *versus* AC-commercial (11 mV; 111 mV) tracks its larger series resistance (0.030 *vs.* 0.022 Ω across the same current range; Fig. S2), indicating greater transport resistance within the porous network.^54–56^

Fig. S2(b) highlights the resistance trends, showing that AC-1.5 maintained stable resistance values of 0.030 Ω across all current densities. In contrast, AC-commercial demonstrated consistently lower resistance, averaging 0.022 Ω. The resistance stability of both samples across varying current densities demonstrate their robustness and reliability for energy storage applications.^56^ Reducing ESR in AC-1.5 therefore requires increasing mesopore volume and/or electrical conductivity (*e.g.*, mild graphitization) while preserving micropore volume, as indicated by the concurrent trends in capacitance and polarization metrics in Fig. 6(f)–(h) and S2.

Fig. 6(g) and (h) evaluate the cycling stability and specific capacitance retention of AC-1.5 and AC-commercial over 7500 charge–discharge cycles at a constant current density of 2 A g^−1^. According to Fig. 6(g), AC-1.5 maintained consistent triangular charge–discharge profiles across cycles 1, 1000, and 7500, reflecting excellent cycling stability and reversible charge–discharge behaviour. AC-1.5 retained 93% of its initial specific capacitance after 7500 cycles, with most capacitance declined occurring within the first 500 cycles, followed by stabilization (Fig. 6(h)). This demonstrates strong resistance to further degradation and highlights the durability of AC-1.5 for long-term applications.

In comparison, AC-commercial retained 100% of its specific capacitance over 7500 cycles, as shown in Fig. 6(h), highlighting its excellent durability under the tested conditions. However, Fig. 6(g) reveals broader and slightly distorted triangular profiles by the 7500^th^ cycle for AC-1.5, indicating increased resistive losses and reduced ionic accessibility. While AC-commercial exhibited no measurable loss in specific capacitance, its lower overall capacitance and diminished performance at higher current densities, as observed in prior analyses, limit its applicability for advanced energy storage applications compared to AC-1.5.

### Further purification at higher temperatures

To further improve the purity of purified AC-1.5, thermal treatment was conducted at various temperatures ranging from 700 to 1500 °C for one hour, targeting the removal of residual oxygen and functional groups. Table 2 summarizes the oxygen analysis and CHNS results, revealing the progressive elemental changes induced by thermal treatment.

**Table 2:** Elemental composition (wt%) of activated carbon samples at different thermal treatment temperatures

Temperature (°C)	N	C	H	S	O
700	3.01	86.07	1.28	ND^a^	14.30
900	2.96	87.82	0.78	ND	12.75
1100	2.16	90.42	0.41	ND	9.90
1300	1.31	98.53	0.20	ND	5.15
1500	0.57	97.37	0.09	ND	1.50

a ND: Not detected.

The oxygen content consistently decreased with increasing temperature, demonstrating the effectiveness of high-temperature treatment in purifying the carbon matrix. At 700 °C, the oxygen content was reduced to 14.3 wt%, reflecting the partial removal of oxygen-containing functional groups, including carboxyl and hydroxyl groups. This trend continued at higher temperatures, with oxygen levels decreasing to 1.5 wt% at 1500 °C, indicating the near-complete elimination of oxygenated impurities. These results highlight the role of thermal treatment in achieving a cleaner carbon surface and enhanced material purity.

The CHNS results corroborate these findings, showing a steady decline in nitrogen and hydrogen content with increasing temperature, consistent with the thermal decomposition of nitrogen- and hydrogen-containing groups. The carbon content increased notably, rising from 86.1 wt% at 700 °C to 98.5 wt% at 1300 °C, signifying a more refined carbon structure. Interestingly, at 1500 °C, the carbon content slightly decreased to 97.4 wt%, which may be attributed to minor structural rearrangements or the loss of volatile impurities. These compositional changes demonstrate the efficacy of high-temperature treatment in producing a highly pure and structurally stable activated carbon.

These findings suggest that purified AC can undergo further refinement through thermal treatment at elevated temperatures, facilitating the removal of residual functional groups and enabling the modification of surface chemistry to meet the demands of specific applications. While such treatments effectively enhance purity by eliminating oxygenated impurities and refining the carbon structure, they may also induce structural changes, including the loss of micropores or alterations in pore connectivity. These changes could impact the specific surface area, pore structure, and overall performance of the material. Thus, a tailored approach is essential to optimize the balance between purity improvement and the retention of critical structural properties, ensuring suitability for advanced applications.

## Conclusion

This study demonstrates the sustainable production of high purity AC from spent coffee ground through optimized alkaline infusion and acid etching. The developed process achieved a highly pure AC material (99.7 wt% purity) with modified structural, compositional, and electrochemical properties compared to AC-commercial, showcasing the potential of biomass waste as a precursor for advanced material applications. Alkaline infusion significantly improved purity and reduced ash content. Biochar, the untreated precursor, exhibited a purity of ∼92 wt% and an ash content of ∼7.8 wt%. Optimized NaOH infusion using 1.5 g NaOH (for 1 g of biochar) increased purity to 99.7 wt% and reduced ash content to <0.3 wt%. Acid etching further enhanced purity by removing residual oxides such as potassium and sodium oxides, as confirmed by XRF analysis. Thermal and structural stability analyses revealed notable improvements in purified AC. FTIR and Raman spectroscopy confirmed the effective removal of oxygen-containing functional groups in purified AC, including hydroxyl and carboxyl groups, and a reduction in defect density. Surface area and porosity analyses highlighted significant enhancements in purified AC. Biochar exhibited a BET surface area of only 1.5 m^2^ g^−1^, with negligible porosity. In contrast, AC-1.5 achieved a surface area of 550 m^2^ g^−1^ and developed a well-interconnected network of micropores (∼1.6 nm) and mesopores, facilitating superior adsorption and charge storage.

Electrochemical performance evaluations demonstrated the superiority of AC-1.5 over AC-commercial. Cyclic voltammetry (CV) results showed that AC-1.5 exhibited a nearly rectangular CV curve, indicating excellent charge storage capacity, while biochar showed limited performance due to minimal active sites. At a current density of 0.5 A g^−1^, AC-1.5 achieved a specific capacitance of 49.0 F g^−1^, significantly outperforming AC-commercial, which exhibited 30.7 F g^−1^. Further thermal treatment enhanced the purity of AC-1.5, reducing oxygen content from 14.3 wt% at 700 °C to 1.5 wt% at 1500 °C, while increasing carbon content from 86.1 wt% to 98.5 wt%, indicating a highly refined carbon structure. These findings highlight the potential of thermal treatment to tailor AC properties for specific applications. This study establishes a novel and scalable approach to convert spent coffee ground into high-purity AC, achieving better performance compared to commercial alternatives. These findings contribute to advancing sustainable material development while promoting resource recovery and circular economy principles. While this study demonstrates the effectiveness of a multi-step purification strategy for producing high-purity activated carbon from spent coffee grounds, it is limited to specific activation and etching conditions. Future work may explore scaling the process, optimizing electrochemical performance in full-cell configurations, and assessing the environmental and economic feasibility for large-scale applications.

## Experimental section

### Materials

Spent coffee ground collected locally was dried at 80 °C to remove moisture and used as the raw material for pyrolysis, purification, and activation. Sodium hydroxide (NaOH, analytical grade), hydrochloric acid (HCl, 32% w/v), and commercial activated carbon (AC-commercial) were procured from Sigma-Aldrich and used without further purification.

### Carbon conversion and chemical activation process

Pure activated carbon (AC) was synthesized through thermal infusion activation with varying NaOH concentrations, followed by acid etching and drying. Initially, a given amount of NaOH (between 0.1–2.0 g) was dissolved in 1.5 to 2.0 mL of deionized (DI) water at 80 °C for 5 to 10 minutes. One gram of carbonized spent coffee grounds was then mixed with the NaOH solution to ensure uniform dispersion. The mixture was infused in a horizontal furnace at 550 °C for 90 minutes under argon flow (1 L min^−1^) to maintain an inert environment as reported in detail elsewhere.^57^ After infusion activation, the sample was sonicated in DI water for 90 minutes to improve dispersion and remove residual NaOH, followed by thorough washing and drying at 120 °C for 3–4 hours.

To further enhance the purity, the dried sample was washed with HCl, designed to eliminate residual inorganic compounds. In this step, a solution of 0.1 M HCl in 10 mL of DI water was prepared, and 0.5 g of the infused carbon was added. The mixture was stirred on a hot plate at 80 °C for 90 minutes. Afterwards, the sample was washed multiple times with DI water until the pH of the rinsed water was neutral, ensuring complete removal of acid residues. Finally, the acid-washed sample was dried in an oven at 120 °C for 3–4 hours, yielding the high-purity AC utilized in this study.

### Characterization techniques

The characterization of the samples was conducted using various advanced techniques to explore their structural, thermal, morphological, and chemical properties.

Fourier transform infrared spectroscopy (FTIR, PerkinElmer Spectrum 100) was employed to identify the functional groups of organic species present in the waste precursors. Measurements were conducted within a wavenumber range of 650–4000 cm^−1^ to investigate the chemical bonds and structural changes during the activation process.

Thermal properties and volatile content were assessed through thermogravimetric analysis (TGA, PerkinElmer TGA 8000). Approximately 10–20 mg of each sample, placed in an alumina crucible, was heated from ambient temperature to 800 °C at a rate of 10 °C min^−1^ under either a nitrogen or an oxygen atmosphere. This analysis provided insights into the decomposition behaviour and thermal stability of the samples.

To determine the crystalline structure and mineralogy, X-ray diffraction (XRD, PANalytical X'Pert Pro) was conducted using Cu Kα radiation within a 2*θ* range of 20–70°. Additionally, semi-quantitative X-ray fluorescence (XRF, PANalytical PW2400 Sequential Wavelength Dispersive) was utilized to estimate the mass percentages of oxide phases in the samples.

Morphological features and semi-quantitative elemental compositions were studied using field-emission scanning electron microscopy (FE-SEM, FEI Nova NanoSEM 450) equipped with energy-dispersive spectroscopy (EDS, Bruker). High-resolution transmission electron microscopy (HR-TEM, JEOL JEM-ARM200F) operating at 200 kV was employed to investigate nanoscale mesoporous structures and polycrystalline nature. TEM samples were prepared by suspending the sample powders in absolute ethanol, followed by 10–15 minutes of sonication and drop-casting of the suspension onto carbon-coated copper grids.

The specific surface area (SSA), average pore diameter, and pore size distribution of the samples were measured using Brunauer–Emmett–Teller (BET, Micromeritics TriStar II 3020 V.3.0) and Barrett–Joyner–Halenda (BJH) methods. Approximately 100–150 mg of each sample was degassed under vacuum at 200 °C before nitrogen adsorption/desorption analysis at −196 °C. These measurements were used to understand the surface properties and porosity of AC.

The chemical structure of the samples was analysed through Raman spectroscopy (Renishaw inVia Raman microscope) using a He–Ne green laser (*λ* = 514 nm) and a diffraction grating of 1800 g mm^−1^. The equipment was calibrated against the silicon peak at 520 cm^−1^ before obtaining Raman spectra in the range of 150–3500 cm^−1^ with a spot size of approximately 4 μm. This technique provided critical information on the graphitic and disordered structures within the carbon matrix.

The purity of all samples was evaluated by analysing their ash content. Approximately 1 g of each sample was weighed and placed in an alumina crucible, followed by combustion in a muffle furnace at 800 °C for 2 hours. After combustion, the residual ash was weighed to determine the ash content, with lower ash percentages signifying higher purity of the AC.

To assess the inorganic elemental composition, X-ray fluorescence (XRF) spectroscopy was utilized. The analysis was performed using a PANalytical AXIOS wavelength-dispersive XRF spectrometer, which provides precise elemental detection ranging from parts per million (ppm) to percent levels.

Elemental composition analyses, including carbon (C), hydrogen (H), nitrogen (N), sulfur (S), and oxygen (O), were conducted to further characterize the samples. CHNS analysis was carried out using an Elementar varioMACRO cube, where samples were combusted at 1150 °C to convert elements into quantifiable gases. Additionally, oxygen content was measured using the Elementar rapidOXY cube, a specialized instrument designed for accurate determination of oxygen in solid materials.

### Supercapacitor fabrication and electrochemical performance analysis

For the fabrication of supercapacitor electrodes, a composite material was prepared by mixing 13.5 mg of activated carbon with 1.5 mg of carbon black (Super P® Conductive, 99+%) to enhance conductivity. To ensure uniform binding, 45 μL of Nafion (5% w/w in water and 1-propanol) was added to the mixture. In addition, an extra amount of isopropanol was added to mix the powder and make the slurry smooth for coating. The resulting composite was homogenized thoroughly and deposited onto a 304 stainless-steel mesh (# 500, 25 *μ*) substrate. The coated substrate was carefully dried overnight on a hot plate at 80 °C and then punched into circular electrodes with a diameter of 15 mm, each containing approximately 10 mg of solid material. Both electrodes were prepared using the same procedure to ensure consistency. A double-electrode coin-cell-shaped supercapacitor was assembled by placing a cellulose-base separator (# 41 Whatman® filter paper) between the two electrodes and adding 100 μL of aqueous 4 M KOH as the electrolyte. The use of an aqueous alkaline electrolyte (4 M KOH) provides high ionic conductivity and stable double-layer formation, which is standard for evaluating the capacitive behaviour of carbon-based electrodes. The device was sealed securely using an electric coin cell crimper to maintain a stable electrolyte environment during subsequent electrochemical analyses.

The electrochemical performance of the fabricated supercapacitor was evaluated at room temperature using a potentiostat/galvanostat (VSP-300, BioLogic, USA). Cyclic voltammetry (CV) and galvanostatic charge/discharge (GCD) tests were conducted over various potential ranges to investigate the charge storage behaviour. Additionally, electrochemical impedance spectroscopy (EIS) was performed at the open circuit potential (OCP) with an amplitude of 10 mV, scanning frequencies from 100 mHz to 100 kHz, to assess the resistance and charge transfer characteristics of the device.

The specific capacitance (*C*_s_) of the electrode materials was determined using both CV and GCD measurements. The specific capacitance from CV was calculated using the equation:12
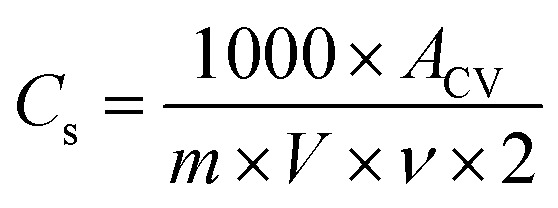
where *A*_CV_ represents the area of the CV curve, *V* is the voltage range, *ν* is the scan rate, and *m* is the mass of activated carbon in the electrode.

From GCD, the specific capacitance (*C*_s_) was calculated using:13
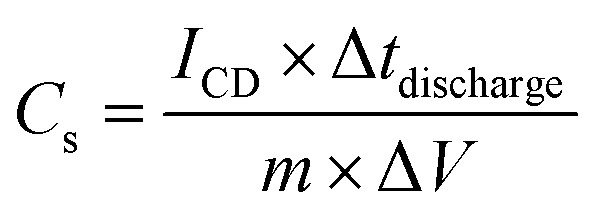
where *I*_CD_ is the discharge current, Δ*t*_discharge_ is the discharge time, and Δ*V* is the potential change during discharge, corrected for any *IR*-drop.

## Conflicts of interest

There are no conflicts to declare.

## Supplementary Material

NA-007-D5NA00658A-s001

## Data Availability

The data supporting this article have been included as part of the supplementary information (SI). Supplementary information: CHNS and oxygen analysis of biochar and purified activated carbon (AC) samples after alkaline infusion and acid etching, differential thermogravimetric data, porosity analysis, adsorption–desorption isotherms, illustrating the structural and functional evolution of purified AC samples. Additionally, electrochemical performance metrics, including *IR* drop and resistance measurements for selected AC samples. See DOI: https://doi.org/10.1039/d5na00658a.
