# Supplementary material for: Greening of coffee waste through its transformation into clean and structurally stable activated carbon for energy storage applications
Source: Nanoscale Adv. 2025 Oct 23;7(24):7930–42. doi: 10.1039/d5na00658a (PMC12548544; doi:10.1039/d5na00658a)
Supplement: NA-007-D5NA00658A-s001 [file NA-007-D5NA00658A-s001.pdf]

Supplementary Information (SI) for Nanoscale Advances.
This journal is © The Royal Society of Chemistry 2025

Supporting Information: Greening of coffee waste through its transformation into clean and structurally stable activated carbon for energy storage applications

Zain Ul Abideen,* Rasoul Khayyam Nekouei, Mohsen Hajian-Foroushani, Samane Maroufi, and Veena Sahajwalla

Centre for Sustainable Materials Research and Technology, (SMaRT), School of Materials Science and Engineering, UNSW Sydney 2052, NSW, Australia

E-mail: zain.abideen@unsw.edu.au

Table S1: CHNS analysis of biochar and purified AC after alkaline infusion and acid etching.

**Sample**	**N (wt. %)**	**C (wt. %)**	**H (wt. %)**	**S (wt. %)**	**Remarks**
Sulfamethazine	20.02	51.4	5.00	11.58	Certified reference material with known elemental composition.
Biochar	3.89	79.33	0.88	ND[a]	Incomplete pyrolysis; moderate carbon content and residual functional groups.
AC-0.1	4.19	87.08	0.95	ND[a]	Highest carbon content; slight nitrogen suggests some residual functional groups.
AC-0.2	3.99	83.51	0.99	ND[a]	Good carbon content; moderate nitrogen and hydrogen levels indicate some functional groups.
AC-0.5	4.00	86.12	1.12	ND[a]	High carbon content; slightly higher hydrogen suggests more surface groups.
AC-1.0	3.68	81.08	0.98	ND[a]	Moderate carbon content; lower nitrogen suggests fewer nitrogen functional groups.
AC-1.5	2.96	83.65	0.91	ND[a]	Good carbon content; reduced nitrogen content indicates improved purity.
AC-2.0	2.23	84.21	0.96	ND[a]	Lowest nitrogen and hydrogen; minimal functional groups and highest purity.

[a] ND: not detected.

Table S2: Oxygen analysis results of biochar and purified AC after alkaline infusion and acid etching.

**Sample**	**Oxygen (wt. %)**	**Remarks**
Acetanilide	12.43	Standard sample with close agreement to reference value (11.84%).
Biochar	16.57	Residual oxygen functional groups due to incomplete pyrolysis.
AC-0.1	12.93	Lowest oxygen content; indicates efficient pyrolysis.
AC-0.2	13.19	Slightly higher oxygen content; moderate functional group reduction.
AC-0.5	14.18	Increasing oxygen content; possible introduction of surface functional groups.
AC-1.0	17.79	Significant oxygen retention; more surface functional groups present.
AC-1.5	17.47	Similar to AC-1, with high oxygen content.
AC-2.0	18.86	Highest oxygen content; suggests many oxygen functional groups or surface reactivity.

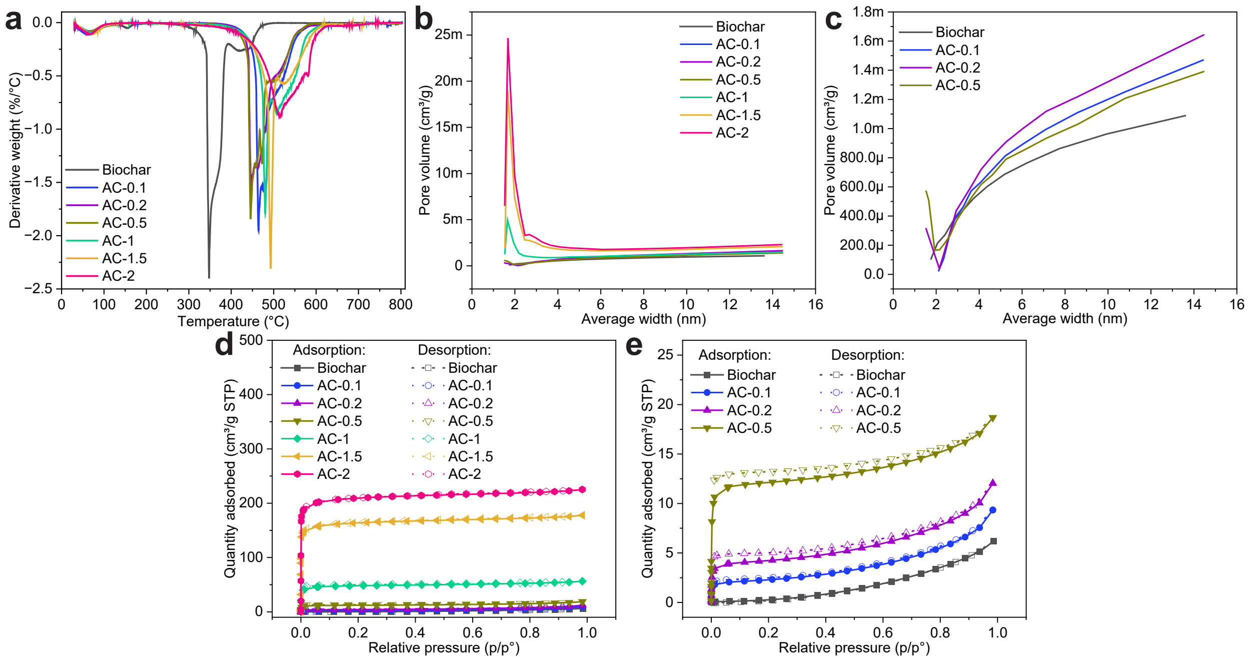

Figure S1: (a)Differential thermogravimetric (DTG) profiles of biochar and purified activated carbon (AC) samples. (b) Pore volume as a function of average pore width for biochar and all purified activated carbon (AC) samples (AC-0.1 to AC-2.0), showing the progression of microporosity and mesoporosity with increasing NaOH concentration. (c) Early stages of pore development for biochar, AC-0.1, AC-0.2, and AC-0.5, highlighting the transition from non-porous biochar to partially activated AC-0.5. (d) Adsorption and desorption isotherms for all samples, with biochar showing Type II isotherms characteristic of non-porous material and activated samples displaying Type IV isotherms indicative of mesoporosity. (e) Adsorption and desorption isotherms of biochar and lower-activation samples (AC-0.1 to AC-0.5)

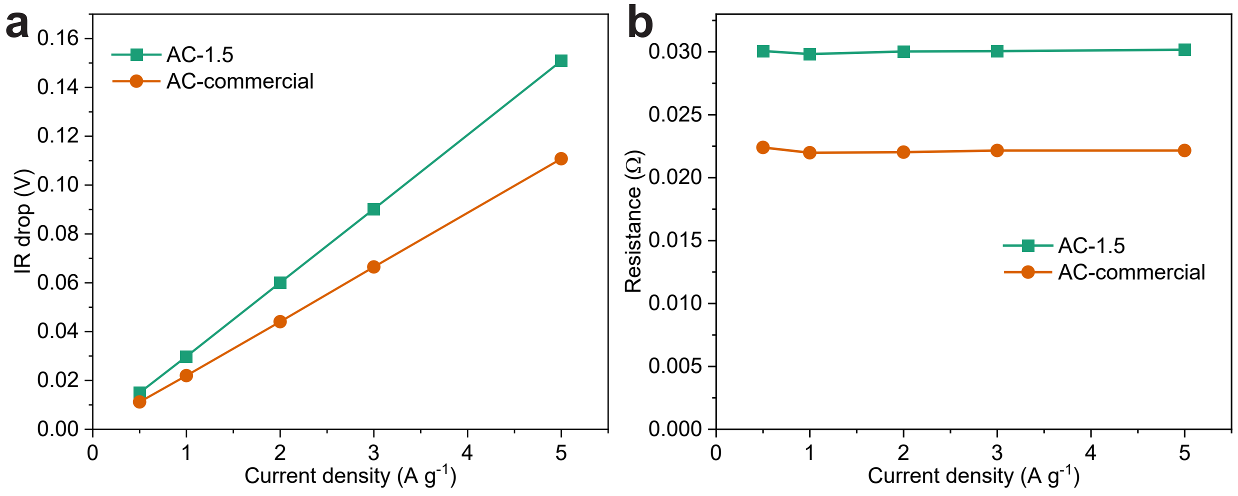

Figure S2: (a) IR drop as a function of current densities for AC-1.5 and AC-commercial. (b) Resistance measurements for AC-1.5 and AC-commercial at varying current densities.
